# Land use change has profoundly altered the process of bacterial community assembly in the northeastern black soil zone

**DOI:** 10.3389/fmicb.2025.1640134

**Published:** 2025-08-26

**Authors:** Ning Sun, Xu Zhao, Fangyuan Liu, Ge Song, Mengmeng Zhang, Fuqiang Song

**Affiliations:** ^1^Engineering Research Center of Agricultural Microbiology Technology, Ministry of Education & Heilongjiang Provincial Key Laboratory of Ecological Restoration and Resource Utilization for Cold Region & Key Laboratory of Microbiology, College of Heilongjiang Province & School of Life Sciences, Heilongjiang University, Harbin, China; ^2^Shenzhen Bolin Environmental Protection Engineering Co., Shenzhen, China; ^3^The Department of Municipal and Environmental Engineering in Heilongjiang Institute of Construction Technology, Harbin, China

**Keywords:** land reclamation, black soil area, soil bacteria, community structure, community assembly

## Abstract

**Introduction:**

Soil microorganisms play a critical role in maintaining ecological functions; however, their responses to grassland reclamation and tillage remain poorly understood. This study aims to investigate the effects of these practices on soil bacterial communities in the Northeast China Black Soil Region.

**Methods:**

We utilized high-throughput sequencing to compare soil bacterial community characteristics between undisturbed grasslands (CK) and reclaimed croplands (F). The analysis focused on assessing structural changes and shifts in ecological strategies of soil bacterial communities under both land-use types.

**Results:**

Our findings revealed that croplands exhibited higher soil pH and elevated concentrations of nutrients such as ammonium nitrogen, nitrate nitrogen, and total phosphorus compared to pristine grasslands. The Average Variation Degree (AVD) indicated that bacterial communities in cropland soils had greater compositional stability. Additionally, dominant bacterial genera, particularly *Sphingomonas* and *Pseudarthrobacter*, were more prevalent in croplands. Using random forest classification modeling, we identified several rare bacterial genera, including *Rhodomicrobium*, *Amycolatopsis*, and *Clostridium*, which, despite each representing less than 1% of the community, played critical roles in shaping microbial community structure. Co-occurrence network analysis showed that reclamation reduced interspecies interactions and significantly decreased network complexity, connectivity, and cohesion (*P* < 0.05). Neutral community assembly modeling further indicated that stochastic processes were more dominant in the assembly of bacterial communities in croplands compared to undisturbed grasslands.

**Discussion:**

This study provides a comprehensive understanding of how grassland reclamation and tillage influence soil bacterial communities in the Songnen Plain black soil region. The findings enhance our ecological understanding of land use changes and offer valuable insights for the sustainable management of black soil resources and ecosystems.

## 1 Introduction

Black soils are known for their high fertility, favorable physical properties, and excellent tillage capacity, making them highly valuable for agricultural production (Chen et al., 2024). Globally, black soil zones refer to large contiguous areas of black soil and cover approximately 7 percent of the Earth’s land surface. However, the extent of these regions has been gradually shrinking in recent years (Liu et al., 2012; Tong et al., 2017). As one of the four main black soil regions globally, Northeast China plays an important role in ensuring food security and ecological stability (Tang et al., 2025). Nevertheless, overexploitation and intensive land use driven by human activities have led to the substantial degradation of black soils and a decline in their ecosystem service functions (Chen et al., 2025). For example, the Songnen Plain, a representative black soil area in Northeast China, is highly suitable for agriculture due to its favorable soil quality and topographic features. In response to growing food demand, large expanses of high-quality grasslands in the region have been converted into arable farmland (Zhang et al., 2021). Such alterations in land use not only result in the depletion of soil fertility (Zhao et al., 2018) but also induce profound changes in soil temperature, humidity, and micro-environmental structure, ultimately leading to a decline in soil carbon sequestration capacity and biodiversity (Liu et al., 2021; Li Z. et al., 2020). Globally, the conversion of grassland ecosystems to agricultural land significantly impacts their ecological functions (Lal, 2004). Grasslands, covering 40% of the Earth’s land surface, are crucial for biodiversity and soil and water conservation (Borer and Risch, 2024; Netsianda and Mhangara, 2025). In China, 18.2% of the cultivated land has been derived from grassland reclamation (Liu et al., 2009), a process that inevitably exacerbates soil erosion and desertification (Heredia-Acuña et al., 2023). Moreover, the extensive application of pesticides and chemical fertilizers during agricultural operations has become the norm (Jiang et al., 2021). This approach significantly changes the soil physicochemical characteristics, such as its pH levels and nutrient composition, and affects the expression of soil enzyme activity (Sagar et al., 2024). This intensive input significantly alters the physicochemical properties of the soil, including pH and nutrient composition, simultaneously impacting soil enzyme activity (Nuruzzaman et al., 2025).

Soil microorganisms are integral components of terrestrial ecosystems, playing crucial roles in nutrient cycling and maintaining environmental balance (Sahu et al., 2019). Among them, certain bacterial taxa contribute significantly to processes such as the regulation of oxygen fluxes and the decomposition of organic matter (Liu et al., 2024). These microbial activities have profound effects on soil quality, fertility, and nutrient availability, thereby supporting long-term soil productivity and ecosystem health (van Midden et al., 2023). Bacteria constitute one of the most abundant and taxonomically diverse groups of microorganisms on Earth and are particularly vital in agricultural systems (Xing et al., 2024). They drive key ecological processes, including nutrient turnover and energy flow within soil ecosystems (Bao et al., 2023). In addition to these functions, soil bacteria enhance resistance to erosion and contribute to improved water and nutrient retention, thereby promoting overall ecosystem stability (Jiang et al., 2025). More importantly, as foundational components of the soil microbiome, bacterial community composition and abundance directly influence soil health and biodiversity (Zheng F. et al., 2024). Land reclamation, however, can significantly alter the structure and function of these microbial communities, leading to downstream effects on ecosystem biodiversity and soil architecture (Santana-Martinez et al., 2024; Schreckinger et al., 2025). As a result, soil bacterial communities are increasingly recognized as sensitive and reliable indicators of land use change and ecosystem functionality (Hermans et al., 2017). Therefore, in-depth investigations into the ecological roles and functional dynamics of soil bacteria are essential for advancing sustainable agricultural practices and conserving natural ecosystems.

In response to the challenges faced in black soil regions, this study was conducted in the Songnen Plain, a representative area in Northeast China known for its black soil. High-throughput sequencing technology was employed as the core research method to systematically investigate the changes in soil physicochemical properties, and the composition, structure, co-occurrence networks, and assembly processes of soil bacterial communities between pristine grassland and croplands with long-term maize cultivation after reclamation. We formulated the following research hypotheses: (1) Reclamation of pristine grassland will significantly affect the *α*-diversity, species composition and symbiotic network structure of soil bacterial communities; (2) Reclamation of pristine grassland will disrupt the original equilibrium between deterministic and stochastic processes in the assembly process of soil bacterial communities; (3) Reclamation of pristine grassland will cause significant changes in soil physicochemical properties, which will then have a significant structural and stability change. This study aims to reveal the mechanism of the impact of reclamation on the black soil ecosystem through an in-depth analysis of the changes in soil physicochemical properties and the structural diversity of soil bacterial communities during the transition from pristine grassland to croplands in the Northeast Black Soil Zone. This study is not only of great significance to the field of ecological research, but also provides directional reference and data support for the conservation and restoration of the Northeast Black Soil Region.

## 2 Materials and methods

### 2.1 Study area

The experimental site is situated in the northern Songnen Plain (46°00′-47°89′N, 123°90′-125°27′E) of northeastern China, within the mid-temperate continental monsoon climate zone. This region is characterized by four distinct seasons, significant temperature variations, and concurrent precipitation and heat. Annual rainfall is relatively low, averaging approximately 560 mm, with 80% occurring between May and October. The mean monthly temperature ranges from −21.6 °C in January to 21.5 °C in July, with an annual average of 1.9 °C, reflecting substantial seasonal temperature fluctuations. The area boasts diverse soil types, primarily including black soil, black calcium soil, meadow soil, and wind-sand soil, with black soil being the most prevalent. Currently, large areas of pristine grassland in this region have been converted into croplands. The region’s flat topography provides favorable conditions for plant growth. Consequently, the area harbors a rich variety of primitive plant species, establishing an excellent natural resource foundation and offering valuable experimental materials and research subjects for scientific investigation and ecological conservation.

### 2.2 Experiment design and soil sample collection

This study was conducted in the northern part of the Songnen Plain, focusing on two distinct land use types: pristine grasslands (CK) and croplands (F) cultivated continuously with maize (*Zea mays* L.) since 1998. The croplands followed an annual management protocol of sowing in May and harvesting in October, with spring ploughing performed mechanically. Fertilizers, including a maize compound fertilizer (N + P₂O₅ + K₂O content ≥40%), were applied at 35–40 kg per acre during planting and an additional 15–20 kg per acre applied in mid-June. A 200-metre isolation buffer separated the croplands from the pristine grassland sample site, ensuring sample independence. The primitive grassland’s surface vegetation comprised a mixed herbaceous plant community, primarily including species such as dogweed, tiger’s tail grass, paintbrush, lobelia, barnyard grass, thousand spirea, small quinoa, grey-green quinoa, amaranth, concave-head amaranth, hogweed, groundnut, and prickly amaranth. The study design incorporated 2 treatments, each with 9 replications, totaling 18 treatment combinations to facilitate robust statistical analyses.

Soil samples were collected in August 2020 for this study. Before sampling, surface vegetation and organic debris were carefully removed from each sample site to ensure collection of the top 0–20 cm of soil. The study area was divided into 25 m × 25 m plots, with a minimum distance of 50 m between adjacent plots to mitigate spatial autocorrelation effects. Within each plot, systematic sampling was conducted using the five-point sampling method. A sterile soil auger was used for sample collection, with careful removal of plant debris, stones, and other extraneous materials. The soil samples from each point were thoroughly homogenized to obtain a final composite sample of approximately 1 kg per plot, resulting in a total of 18 soil samples. Stringent anti-contamination measures were implemented throughout sample collection, transport, and storage. All sampling tools were thoroughly decontaminated before use, and sterile polyethylene self-sealing bags were organized to store the samples the samples. While collecting samples, the top layer of loose soil and vegetation was cleared away to avoid contamination. Initial screening of the soil was conducted on-site through sieving, which helped remove stones, plant roots, and other unwanted materials (Wang et al., 2024). During transport, soil samples were segregated and stored based on their characteristics and intended use to prevent cross-contamination. To maintain sample integrity, soil samples were briefly stored at 4 °C to retard microbial growth and chemical reactions. For subsequent analyses, a portion of the samples was air-dried, ground, and passed through a 100-mesh sieve for physicochemical property determination; another portion was stored at −4 °C for soil enzyme activity assessment; and the remaining samples were stored at −80 °C for future extraction and analysis of total soil DNA.

### 2.3 Determination of soil physicochemical properties

The air-dried soil samples, once ground to pass through a 100-mesh sieve, were utilized for systematic chemical property analysis. To measure soil pH, we proceeded as follows: precisely weighed 10.0 grams of the air-dried, sieved soil and placed it in a 50 mL beaker. Added 25 mL of deionized water, maintaining a soil-to-water ratio of 1:2.5, stirred continuously for 1 ~ 2 min using a glass rod, and then left it to equilibrate for 30 min. We then determined it using a pH meter of type FE20 (Mettler Toledo, Shanghai, China) (Wang et al., 2018). Soil electrical conductivity (EC) was measured using a FE38 model conductivity meter from Mettler Toledo, Shanghai, China. The soil organic matter (SOM) content was accurately determined using the high-temperature exothermic potassium dichromate oxidation-volumetric method as outlined by Zhou et al. (2014). The soil total nitrogen (TN) content was assessed through the classical Kjeldahl method. For estimating ammonium nitrogen (NH₄^+^-N) and nitrate nitrogen (NO₃^−^-N) in the soil, the procedures described by Wang et al. were followed as a reference. First, we did the extraction using 2 M KCl solution. Next, we measured the ammonium content using indophenol blue colorimetry with the A580 UV spectrophotometer (Aoelab, Shanghai, China) (Wang et al., 2018). For available phosphorus (AP), we used the sodium bicarbonate method with the same A580 instrument. This method has two main advantages: high accuracy and simple operation. To measure available potassium (AK), we extracted samples with ammonium acetate and read results using the AP1500 flame photometer (Aoelab, Shanghai, China). This common method gives precise soil potassium measurements. For total phosphorus (TP) and potassium (TK), we followed Schut’s method (Schut and Reymann, 2023). First, we moistened soil samples with alcohol. Next, added NaOH to cover the soil. Then heated in two stages: 400 °C for 15 min, then 700 °C for 30 min. After cooling, we treated the samples with 5% sulfuric acid. Finally, did the final measurements.

### 2.4 DNA extraction

Based on the detailed steps listed in Table 1, the total microbial DNA in the soil samples was extracted, and then the extracted DNA samples were subjected to rigorous quality testing and concentration determination to ensure that they met the technical requirements for high-throughput sequencing analysis.

**Table 1:** DNA extraction and detection.

Procedure	Operating method
DNA extraction	OMEGA Soil DNA Kit
DNA quality testing	0.8% agarose gel electrophoresis
Determination of DNA concentration	Ultraviolet spectrophotometer (Nanodrop 2000)

The hypervariable V3-V4 segments of bacterial 16S ribosomal RNA genes were amplified through polymerase chain reaction employing universal primer pair 338F (5′-ACTCCTACGGGGAGGCAGCA-3′) and 806R (5′-GGACTACHVGGGGTWTCTAAT-3′). The PCR reaction system was as follows: The 25 μL amplification mixture consisted of 5.00 μL 5 × reaction buffer, 5.00 μL 5 × GC enhancer, 2.00 μL of 2.5 mM dNTP mix, 1.00 μL each of forward and reverse primers (10 μM concentration), 2.00 μL genomic DNA template, 8.75 μL ultrapure water, and 0.25 μL Q5 high-fidelity DNA polymerase. Thermal cycling conditions were initiated with 2 min of pre-denaturation at 98 °C, followed by 30 amplification cycles, which comprised three sequential steps: 15-s denaturation at 98 °C, 30-s primer annealing at 55 °C, and 30-s elongation at 72 °C, concluding with a 5-min terminal extension at 72 °C, as per the method described by Atashpaz et al. (2010) products were sent to Shanghai Meiji Biologicals Co., Ltd., where they were analyzed by double-end sequencing using the Illumina MiSeq platform.

### 2.5 Data analyses

QIIME (V1.7.0) was utilized to process the raw data (Heidrich et al., 2021). Trimmomatic software (Bolger et al., 2014) was employed to remove low-quality sequences, with parameters set to exclude sequences shorter than 200 bp, those with a Q-value below 30, sequences containing ambiguous bases, mismatches of more than one primer base, and sequences unrecognizable by Barcode. Chimeric sequences were detected and removed using UCHIME software, referencing the ‘RDP Gold’ database (Schloss et al., 2011). USEARCH software (Zhou et al., 2024) clustered the operational taxonomic units (OTUs) of non-repetitive sequences at a 97% similarity level, generating representative OTU sequences. These OTU representative sequences underwent taxonomic analysis and annotation using the SILVA database, producing clustering information for each sample OTU. Data normalization was performed using the minimum number of sequences method, with 30,000 sequences per sample. Principal component analysis (PCA) characterized the natural distribution among samples, analyzing community composition at the genus level using R software. Referring to the method by Xun et al., the Average Variance Degree (AVD) was used to assess bacterial community stability (Xun et al., 2021).

First, we used an analysis of variance (ANOVA) from R’s “vegan” package to compare differences in soil properties and bacterial diversity between treatments. Next, we calculated bacterial diversity (alpha diversity) for each sample using the “vegan” package, employing Mutlu’s method (Mutlu et al., 2017). We then performed an NMDS analysis based on Bray-Curtis distances to investigate differences between samples in the “vegan” environment (*β* diversity) (Taguchi and Oono, 2005). For visualization, we created Circos plots using the “RCircos” software package (Zhang et al., 2013). To find key bacteria after tillage, we created random forest models using the following R packages: “randomForest,” “psych,” “reshape2,” and “ggplot2” (Gustavsson et al., 2022). We used the relative abundance data of bacterial genera to define the “structure” of soil bacterial communities. The community structure is mainly described by the relative abundance of different bacterial genera in each sample. Finally, we analyzed the relationships between bacteria using the “igraph” package (Ju et al., 2016). Co-occurrence network visualization maps were created using Gephi.^1^ To assess the empirical network’s characteristics, random networks were simulated with the same number of nodes and edges, employing the Erdös-Réyni model. The topological indices of these simulated networks were summarized over 999 iterations, providing insights into the empirical network’s structure and properties by allowing comparisons against the randomized network behaviors to determine significantly non-random network properties (Hoffman and Holzer, 2019). Referring to the method by Hernandez et al. (2021) Network analysis was conducted within the R statistical environment (v4.2.1) to quantify topological characteristics including module cohesion indices and system resilience parameters within microbial co-occurrence networks, following established computational frameworks. Through implementation of the igraph package (v1.3.5), network deconstruction was performed via node-specific subgraph extraction algorithms, enabling targeted topological examinations. Microbial community assembly processes were decoded using the Sloan stochasticity model through CRAN-hosted analytical modules (Chen et al., 2019). Multivariate visualization techniques included: (1) LDA Effect Size (LEfSe) profiles generated via ggplot2 (v3.4.0), (2) taxonomic overlap diagrams constructed with ggvenn (v0.1.9), and (3) phylogenetic heat-tree representations produced using metacoder (v0.3.3) to elucidate lineage-specific associations (Coller et al., 2019). Environmental factor-microbe interactions were determined through Mantel correlation matrices with 999 permutations. Between-group variance significance was statistically validated in SPSS (v27.0) employing Kruskal-Wallis H-tests with Benjamini-Hochberg correction, as detailed in methodological benchmarks (de León et al., 2011).

## 3 Results

### 3.1 Soil properties

The reclamation of pristine grassland significantly altered the soil chemical properties (Supplementary Table S1; *p* < 0.01). In the reclaimed farmland soil, the pH value, nitrate nitrogen (NO_3_^−^-N), ammonium nitrogen (NH_4_^+^-N), total phosphorus (TP), and available phosphorus (AP) contents were all elevated compared to the pristine grassland. However, there was no significant difference in soil EC.

### 3.2 Soil bacterial diversity and composition

**Figure 1:**
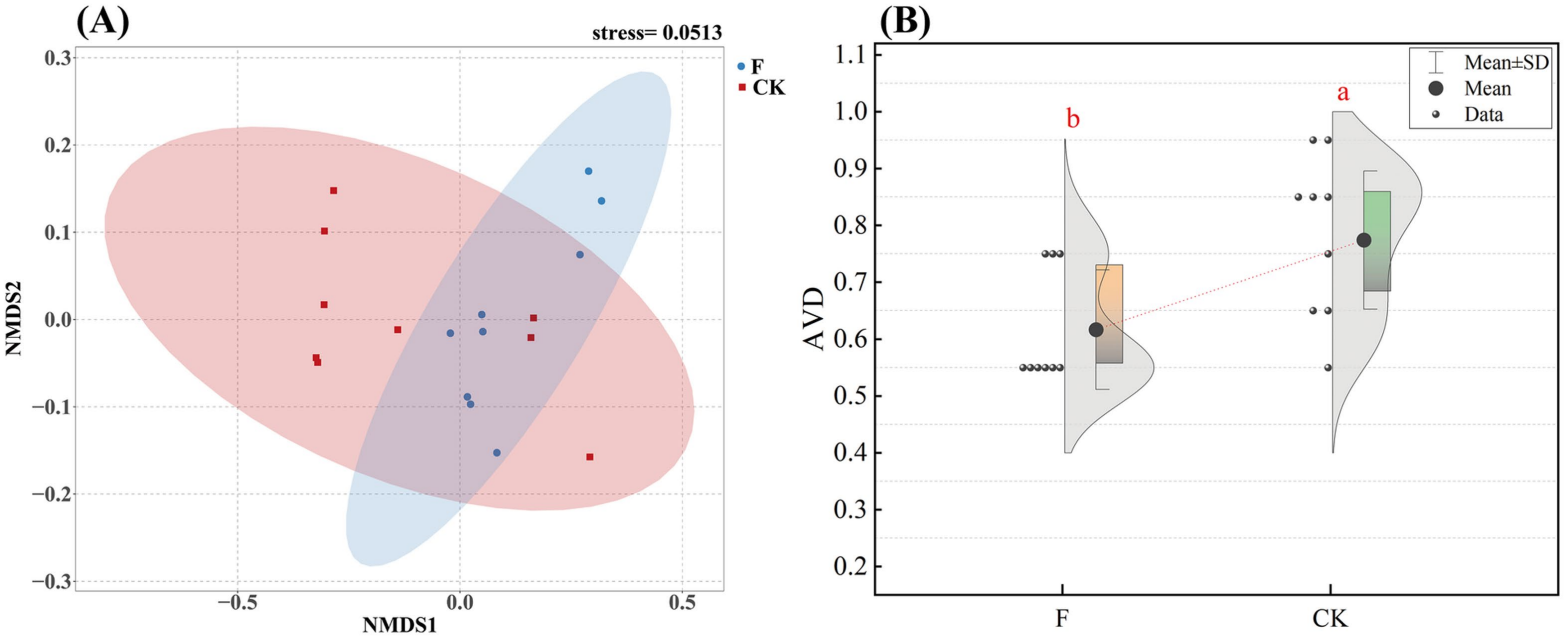
**(A)** Nonmetric multidimensional scaling (NMDS) analyses were conducted to evaluate the soil bacterial communities in croplands (F) and pristine grassland (CK). The stress value indicates the error between the original data distances and the distances in the low-dimensional space derived from NMDS; **(B)** Distribution of average variability (AVD) in the two treatments (F and CK). The dots in the figure show the specific distribution of data points in each group. Shaded areas indicate the density of distribution of the data. Different letter labels indicate significant differences (*p* < 0.05).

Our research showed that reclamation versus ploughing of pristine grassland significantly influenced the variance distribution of soil bacterial communities (Supplementary Figure S1). Comparative analysis of soil bacterial community alpha-diversity under the two treatments revealed that soil bacterial community alpha-diversity indices such as Chao1, ACE, and Shannon index were significantly higher in the F treatment than in the CK treatment (Supplementary Table S2; *p* < 0.01). To further explore the differences in *β*-diversity between soils with different treatments, we employed NMDS based on the Bray-Curtis distance (Figure 1A). The results indicated significant differences in the *β*-diversity of soil bacterial communities between the pristine grassland and the croplands. The stability of the bacterial community between the two treatments was evaluated by the average variation degree (AVD) index, and the results showed that the AVD value of the F treatment was significantly lower than that of the CK treatment, which indicated that the bacterial community in the farmland had a stronger stability (Figure 1B).

**Figure 2:**
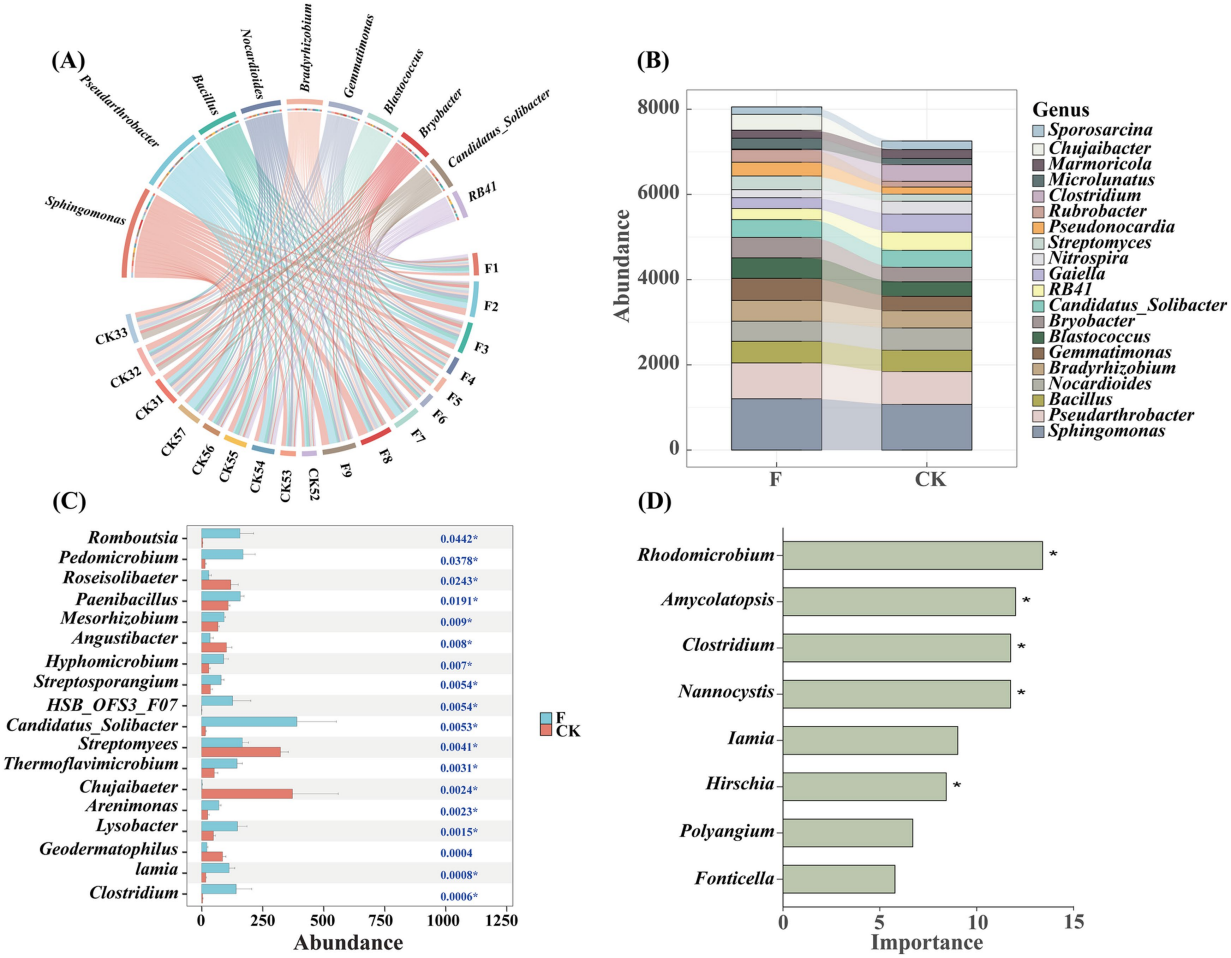
**(A)** Circos showed the top 10 genera of soil bacterial communities in farmland (F) and pristine grassland (CK). The width of the line represents the size of the abundance; **(B)** Stacked charts represent the soil bacterial genera in croplands (F) and pristine grassland (CK). The Random Forest classification modeling visually showed the dominant genera of bacteria in croplands (F) and pristine grassland (CK); **(C)**The significance plot was used to assess differences in bacterial genera between treatment groups. In this plot, different colored bars indicate the various treatment groups, with red representing the CK treatment and blue representing the F treatment. The horizontal axis shows the percentage of genera in each treatment group, while the vertical axis lists the names of the different genera; **(D)** The importance of random forest variables, derived through a categorical algorithm, is highlighted in predicting rare bacterial genera in soils. In this visualization, green bars denote the variables that were selected using the categorical algorithm. These bars illustrate the contribution of each variable to the model, emphasizing their role in the accurate prediction of rare bacterial genera in soil samples. Significant differences of asterisk were marked with * (*p* < 0.05).

The Circos plot (Figure 2A) illustrated that the predominant soil bacterial genera across all samples are *Sphingomonas* and *Pseudarthrobacter*, with these genera exhibiting greater enrichment in samples under F treatment. Stacked plots revealed significant changes in the composition of soil bacterial communities at the genus level following reclamation and tillage activities. Among the top 20 most abundant genera, a greater number demonstrated increased abundance under the F treatment (Figure 2B). A significance plot test was employed to assess differences in bacterial genera between treatment groups (Figure 2C), revealing significant variations between the bacterial genera of the two treatments. Among the differential genera, *Clostridium* demonstrated the highest abundance in the CK treatment, while *Chujaibacter* showed greater abundance in the F treatment. Moreover, a random forest classification model was utilized to identify microbial groups most significantly influencing soil microbial community structure. The findings indicated that some less abundant genera, such as *Rhodomicrobium* and *Amycolatopsis*, despite contributing less than 1% to the total abundance, played crucial roles in the model (Figure 2D), thereby underlined the potential significance of low-abundance genera within the soil microbial community.

**Figure 3:**
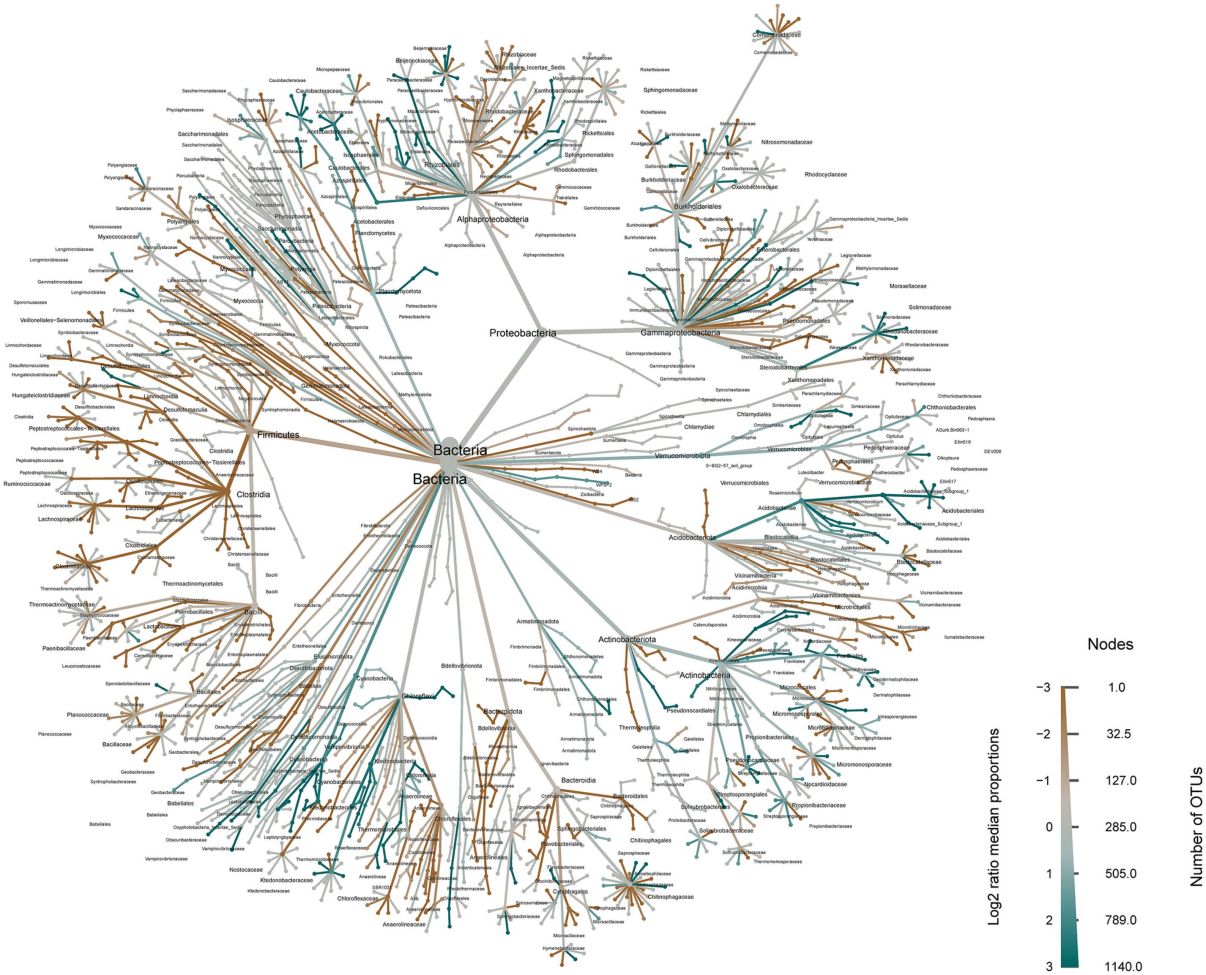
Heat trees illustrate the mean proportion of bacterial communities. Nodes signify each taxonomic level from kingdom (bacteria, center) to species (at branch tips). The width of nodes and branches indicates the average proportion of that taxon in samples within relative groups. Node size reflects the count of taxa present, while color intensity indicates proportions relative to all bacterial samples. The color represents the log^−2^ ratio of median proportions of reads found at each body site, with only significant differences highlighted. Significance is determined using a Wilcoxon rank-sum test with Benjamini-Hochberg correction for multiple comparisons.

The LEfSe (linear discriminant analysis effect size) analysis revealed significant differences in the relative abundance of soil bacteria between the primitive grassland and the ploughed field following reclamation (Supplementary Figure S2). Subsequently, we visualized the average proportions of bacterial communities using a heat-tree diagram in the evolutionary classification of bacteria. This visualization demonstrated that Proteobacteria, Firmicutes, and Acidobacteriota exhibited the highest proportions among bacterial phyla. Further analysis of the variability confirmed the significant impact of reclamation and ploughing on the structure of the soil bacterial community (Figure 3).

### 3.3 Soil bacterial co-occurrence network

**Figure 4:**
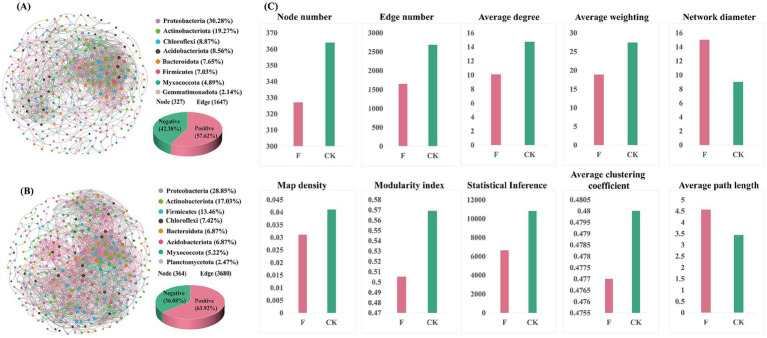
**(A)** The co-occurrence network of croplands (F)showing operational taxonomic units (OTUs) as different dots. The size of the dots is proportional to their degree within the network. Different colors represent different phyla of bacteria. Blue lines indicate positive correlations between OTUs, while pink lines indicate negative correlations. The accompanying pie chart illustrates the ratio of positive to negative correlations within the network. **(B)** The co-occurrence network of the pristine grassland (CK), depicted similarly to the network in panel **(A)**. The network structure and relationships are visualized with the same color scheme for bacterial phyla, and types of correlations are represented by blue (positive) and pink (negative) lines. The pie chart for this network similarly represents the ratio of positive to negative correlations within the network. **(C)** Comparative analysis of network topological attributes between F and CK treatments, including node number, edge number, average degree, average weighting, network diameter, map density, modularity index, statistical inference, average clustering coefficient, and average path length. These metrics provide insights into the complexity and connectivity of the bacterial community networks under different land-use treatments.

To delve into the alterations in bacterial community structures between croplands (Figure 4A) and pristine grassland (Figure 4B), we conducted a co-occurrence network analysis on the top eight abundant phyla. The findings revealed disparities in the top eight phyla between croplands and pristine grassland. Although the first seven phyla remained consistent across both treatments, with Proteobacteria and Actinobacteriota consistently ranking in the top two by abundance in both groups, the order of abundance for other phyla experienced marked changes. Notably, the abundance of *Firmicutes* underwent the most significant alteration in the F treatment, when compared to the CK treatment.

Further analysis of the co-occurrence network topology (Figure 4C) included metrics such as nodes, links, intermediacy concentration, and modularity. The results revealed that the co-occurrence network of the pristine grassland (CK) comprised 364 nodes and 2,680 edges, while that of the croplands (F) consisted of 327 nodes and 1,647 edges. The CK treatment exhibited a higher network graph density (0.041) and average degree (14.725) compared to the F treatment (network graph density of 0.031 and average degree of 10.073), while the average clustering coefficients of the two were similar. These findings suggested that reclamation and ploughing reduced the complexity of the co-occurrence network. Moreover, the positive correlation ratio in the CK treatment (63.92%) was significantly higher than in the F treatment (57.62%), further illuminating the impact of reclamation and ploughing on the structure of the soil bacterial communities’ co-occurrence network.

**Figure 5:**
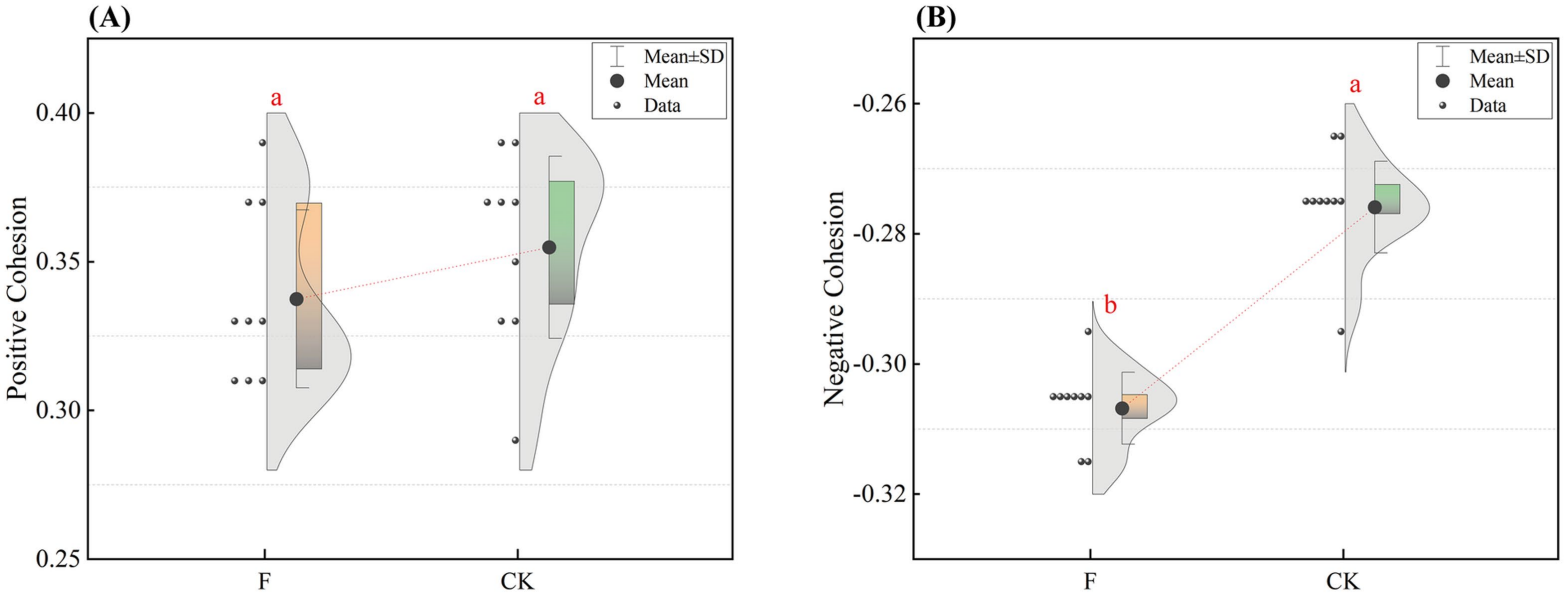
Comparison of Positive and Negative Cohesion of bacterial networks. Panel **(A)** shows the difference in positive cohesion between F and CK treatment. Panel **(B)** shows the negative cohesion. Different letter labels indicate significant differences, *p* < 0.05.

After this we calculated the difference in positive and negative cohesion of bacterial co-linear networks between the two treatments (Figure 5). As can be seen from Figure 5, the positive cohesion of the bacterial co-occurrence network was higher but the difference was not significant in the CK treatment compared to F, while the negative cohesion of the bacterial co-occurrence network in the CK treatment was significantly higher than that in the F treatment (*p* < 0.05). The results indicated that reclamation and ploughing had a significant effect on the negative cohesion of the bacterial community, but had the less effect on the positive cohesion.

### 3.4 Soil bacterial community assembly processes

**Figure 6:**
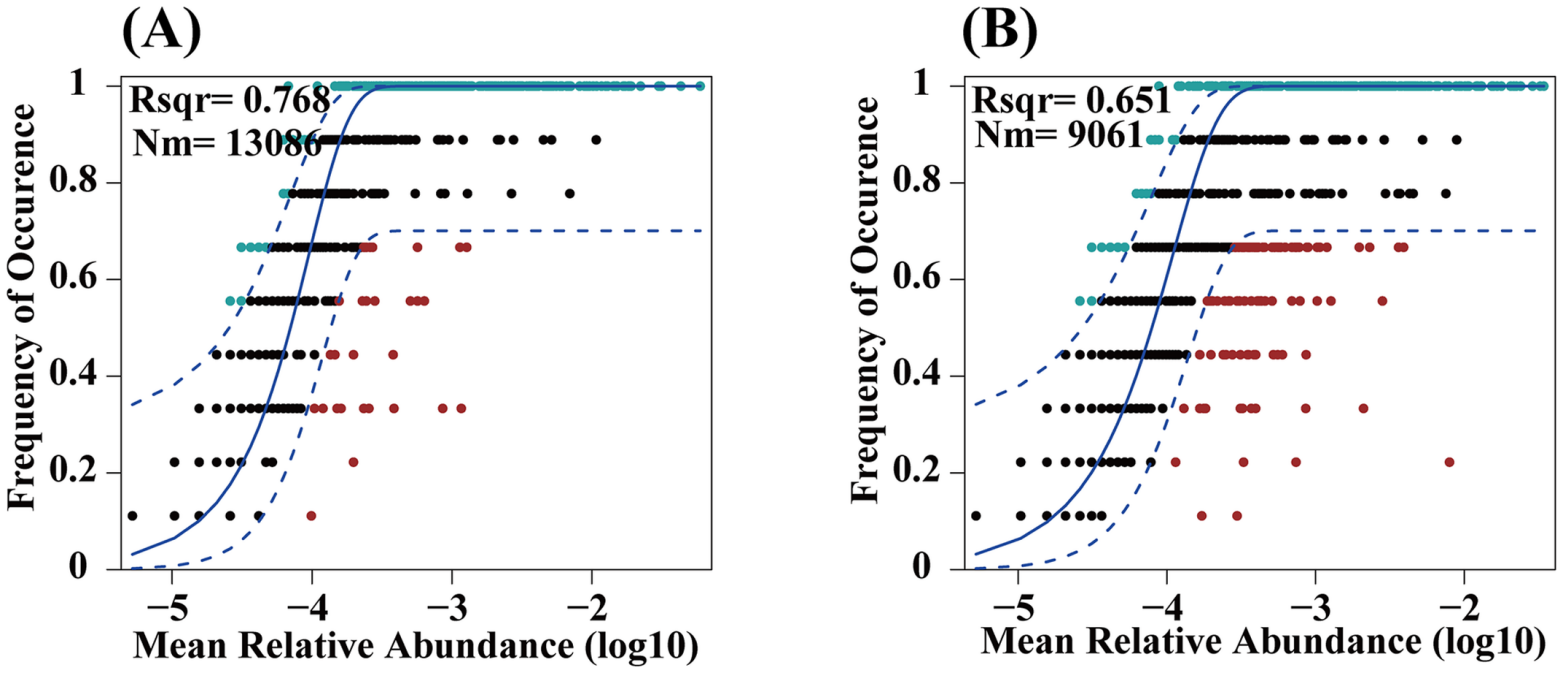
Analysis of soil bacteria using Neutral Community Models (NCM) in croplands **(A)** and pristine grassland **(B)**. **(A)** The plot shows the relationship between mean relative abundance (log10) and frequency of occurrence, with an R² value of 0.768 and a metacommunity size and migration rate product (Nm) of 13086. The solid blue line represents the best fit to the NCM, while the dashed blue line indicates the 95% confidence intervals. **(B)** This plot illustrates a similar relationship with an R² value of 0.651 and an Nm of 9061. The markers represent operational taxonomic units (OTUs), with those within the confidence intervals considered neutrally distributed.

The assembly processes of bacterial communities in croplands and pristine grasslands were assessed using the Sloan neutral community model (Figure 6). The results showed that land use practices altered the assembly processes of soil bacteria, mainly by influencing the equilibrium between deterministic and stochastic processes. Both pristine grassland (CK) and ploughed field (F) showed higher sensitivity to stochastic processes. Both CK treatment and F treatment were more influenced by stochastic processes, but compared to pristine grasslands, the bacterial community in cropland soil is more significantly affected. Additionally, the bacterial communities in farmland soil have a stronger dispersal ability, leading to a more uniform distribution of species between communities.

### 3.5 Factors influencing the soil bacterial community composition

**Figure 7:**
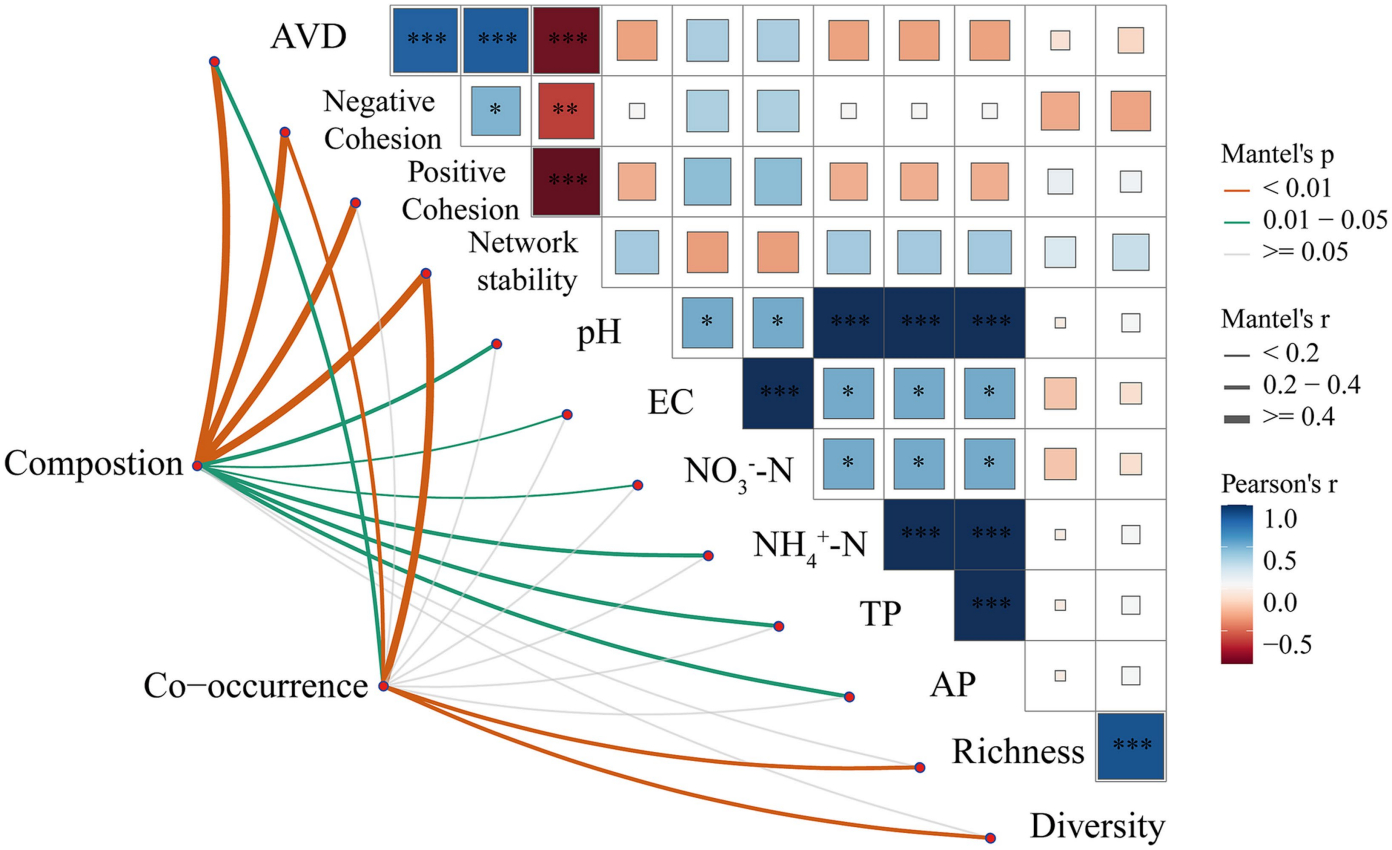
Mantel and Pearson correlation analyses of bacterial community composition, co-occurrence patterns with soil properties, and community diversity. The left part shows the results of Mantel tests between bacterial community characteristics (Composition and Co-occurrence) and environmental factors, Network stability = |Negative Cohesion| / Positive Cohesion, where line colors and types indicate different levels of *p*-values (orange: *p* < 0.01; green: 0.01–0.05; grey: *p* ≥ 0.05), and line thicknesses indicate the Mantel’s r-value magnitude. The right matrix section shows the Pearson correlations between the different factors, with the color shade indicating the strength of the correlation and the color distinguishing between positive (blue) and negative (red) correlations. Asterisks in the matrix indicate the level of significance (* *p* < 0.05, ** *p* < 0.01, *** *p* < 0.001).

An investigation into the composition and co-occurrence patterns of bacterial communities, in correlation with environmental factors, was conducted through Mantel analysis. As shown in Figure 7, Mantel analysis showed that the AVD index, negative cohesion, and stability of the network were all significantly correlated with the composition of the bacterial community, with both composition and co-occurrence. Composition was significantly correlated with positive cohesion as well as with a variety of soil chemical factors such as pH, EC, NO_3_^−^-N and NH₄^+^-N, but co-occurrence was not significantly correlated with these indicators. Meanwhile, richness and diversity showed a more significant correlation with co-occurrence and a non-significant correlation with composition. Pearson correlation analysis indicated a significant negative correlation between network stability and positive and negative cohesion, as well as AVD, while a significant positive correlation was observed between richness and diversity.

## 4 Discussion

### 4.1 Bacterial community structure and composition under changing land use patterns

Changes in land use patterns and inappropriate agricultural practices have the potential to trigger profound changes in the soil environment, including shifts in physical properties and nutrient content, impacting the *α*-diversity of soil bacterial communities. This experimental study focused on undisturbed pristine grasslands and croplands as two treatment groups, aiming to investigate the potential effects of reclamation and ploughing activities on soil bacterial community structures in the Songnen Plain. The results aligned with our initial hypothesis, showing significant differences in α-diversity between the two treatments, with all diversity indices higher in cropland soils than in pristine grasslands (Supplementary Table S2). This suggests higher bacterial species richness and diversity levels in reclaimed and ploughed soil environments. Furthermore, NMDS (non-metric multidimensional scaling analysis) based on Bray-Curtis distance confirmed significant variability in bacterial community structure between the two treatment groups (Figure 1). To explore the stability of the bacterial community, we used average variability (AVD) to evaluate the stability of the bacterial community by referring to the method of Niu et al. (2017). In our study, the mean variance (AVD) value of the F treatment was significantly lower than that of the control (CK) treatment. We speculate that a single factor may not dominate the difference in the stability of the bacterial community, but rather the result of the interaction between environmental interference and nutrient redistribution. The farmland environment where the F treatment group was located originated from the interference of grassland. This interference process prompted the bacterial community to undergo a screening mechanism to adapt to the new environment, thereby enhancing the stability of the community. Nutrient redistribution, as an important regulatory factor in the ecosystem, after the application of nitrogen, phosphorus, potassium and other fertilizers in farmland, can provide more resources to support the growth of certain bacterial populations, thereby promoting functional redundancy and stability within the community (Allison and Martiny, 2008). It not only regulates the growth rate of microorganisms but may also change the spatial distribution of resources. It affects the composition of bacterial communities and their adaptive relationship with the environment (Sentis et al., 2021). It can be seen from this that the synergistic effect between environmental disturbance factors and nutrient redistribution mechanisms has significantly enhanced the adaptability of microbial communities to external environmental stress. This dynamic interaction relationship enables the F treatment group to maintain a more stable community structure and ecological function stability when facing environmental disturbances (Zhang et al., 2018).

Changes in land use significantly alter bacterial habitats, impacting nutrient availability and environmental conditions. Fertilizer and herbicide use in croplands enriches soils with nutrients, fostering bacterial colonization (Liu et al., 2025). Differences in nutrient inputs between grasslands and croplands result in homogenous carbon and nitrogen in croplands, enhancing bacterial competition in similar niches (Wang and Kuzyakov, 2024). Simultaneously, to adapt to the changing environment, bacteria undergo functional differentiation and ecological niche rearrangement, inevitably leading to changes in bacterial communities (Wang and Zou, 2024). Changes in bacterial community structure (*β*-diversity) are also closely related to anthropogenic impacts. For instance, excessive application of ammonia fertilizer may lead to excessive proliferation of ammonia-oxidizing bacteria, which in turn inhibits the growth and reproduction of other bacteria (Zhao et al., 2025). Additionally, herbicide use may selectively eliminate certain competitively advantageous bacteria, allowing others to overpopulate, leading to a remodelling of microbial community structure (Bhardwaj et al., 2024). Changes in above-ground plant communities significantly impact soil bacterial communities. The diverse herbaceous vegetation in pristine grasslands contrasts with maize-dominated reclaimed lands. This shift alters plant residues, influencing soil properties and, consequently, the distribution of soil bacterial communities. Zheng B. et al. (2024) showed that changes in plant type significantly change soil microbial types. They found more fungi in some forest soils and also found a surprisingly high number of bacterial species in simple lawn soils. Other studies have confirmed that changing land use can have a large impact on soil microorganisms (Xiao et al., 2021). For instance, land clearing and long-term ploughing can disrupt soil structure, and this compromises the ecological distribution of microorganisms and nutrient availability. D’Acunto et al. (2016) found soil microbial diversity positively correlates with pH, with ecosystems acidifying under natural disturbances, aligning with our findings on apoplastic matter, organic accumulation, and pH correlations.

In this study, we found that *Sphingomonas* and *Pseudarthrobacte*r were particularly abundant across all samples, with higher abundance observed in the F treatment group compared to the CK group (Figure 2B). *Sphingomonas* is a Gram-negative bacillus that is found widely in soil and the areas around plant roots. Earlier studies have shown that *Sphingomonas* can effectively break down pesticides and harmful substances in soil, and its metabolites can help plants grow. So, it is often used as a biofertilizer or biopesticide because of these useful properties (Li et al., 2017; Mizukami-Murata et al., 2016). In contrast, *Pseudarthrobacter* is a Gram-positive bacterium that can break down some pesticides. Also, it helps fix nitrogen and stops harmful plant bacteria from growing (Chai et al., 2023). The large amounts of *Sphingomonas* and *Pseudarthrobacter* in cropland soil likely come from pesticide use. These chemicals killed many microbes, but pesticide-breaking bacteria survived and spread because they are more resistant. In addition, bacteria involved in nutrient cycling, such as *Gaiella* and *RB41*, were more prevalent in the CK group. Pristine grasslands have evolved complex plant and microbial communities over long periods, leading to high ecosystem stability. This complexity enhances resistance to disturbances and promotes the production of rich apoplastic materials and root secretions, vital for sustaining stable soil nutrient cycles.

In addition to focusing on the dominant bacterial genera in the different treatments, this study highlights the important contribution of low-abundance genera in soil bacteria. In constructing the Random Forest classification model, we found that only one of the five genera that had a significant effect on soil bacterial community structure was among the top 20 genera in terms of abundance. The results of the model analysis showed that *Rhodomicrobium*, *Amycolatopsis*, *Clostridium,* and *Nannocystis* were the key genera that shaped the soil bacterial community structure (Figure 2D), and they played a crucial role in soil bacterial community construction, which might be closely related to their functions in nutrient cycling. Specifically, both *Rhodomicrobium* and *Clostridium* are involved in nitrogen cycling processes. According to Conners et al. (2024), *Rhodomicrobium* is a photosynthetic autotrophic bacterium that can use NH_4_Cl or N_2_ as a nitrogen source for nitrogen fixation, converting nitrogen into a form that can be absorbed and utilized by plants (Conners et al., 2024). However, *Clostridium*, as an anaerobic bacterium, can efficiently decompose organic matter and fix nitrogen under anaerobic conditions. Experimental studies by Meng et al. (2024) showed that the use of *Clostridium* as a microbial agent could reduce the loss of nitrogen in the composting process of food waste and accelerate the decomposition of compost to achieve the synergistic conversion of carbon and nitrogen in compost. Thus, *Rhodomicrobium* and *Clostridium* significantly drive soil nutrient cycling, enhance soil fertility, support plant growth, and maintain soil bacterial diversity and stability.

Whereas, the role of *Amycolatopsis* and *Nannocystis* is more on the inhibition of pathogenic bacteria. Gopalakrishnan et al. (2019) in their study on *Amycolatopsis* found that the fungus not only promotes the growth of sorghum, but also has resistance to sorghum charcoal rot. The study of Alipour Kafi et al. (2021) also showed that *Amycolatopsis* has an anti-pathogenic effect on cucumber grown in greenhouses, demonstrating significant potential for the development of biocontrol agents. On the other hand, the experiments of Visioli et al. (2018) found that *Nannocystis* may have a strong inhibitory effect on *Aspergillus flavus*, stimulate the growth of rare bacterial taxa in maize rhizosphere soil, and promote maize growth and development. The inhibitory effect of these genera on pathogenic bacteria is not only a reflection of their biological properties, but also one of the important mechanisms by which they occupy a key position in the soil bacterial community and participate in shaping the community structure. By inhibiting the growth of pathogenic bacteria, they provide a more favorable environment for other beneficial microorganisms, thus promoting the diversity and stability of soil bacterial communities. This ecological niche competition and dominant species promotion effect not only profoundly affects the composition and dynamics of soil bacterial communities, but also provides valuable microbial resources for sustainable agricultural development and soil ecological restoration.

### 4.2 Bacterial co-occurrence networks under changing land use patterns

Soil bacterial communities interact through cooperation and competition. We examined these interactions by focusing on the top 8 most abundant bacterial phyla and constructing interaction networks. The results of the co-occurrence network analysis showed that both connections (edges) and bacteria (nodes) of the soil bacterial network decreased in the croplands compared to the pristine grasslands. These changes make the bacterial communities are simpler and less connected overall (Figure 4). This supports findings by Qiu et al. (2024) that more complex networks are generally more stable. To better illustrate the stability of the co-occurrence network, we further calculated the positive and negative cohesion of the F and CK treatments, and the results showed that both positive and negative cohesion of the CK treatment were higher than that of the F treatment, with the negative cohesion showing a significant difference. Generally, positive cohesion refers to the collaborative relationship between microorganisms, while negative cohesion refers to the competitive relationship between microorganisms, and networks with high cohesion are generally more stable, which is consistent with our previous prediction (Zhang et al., 2024). The complexity of co-occurring networks often serves as an indicator of bacterial community resistance to external disturbances and community stability. Cohesive networks are typically more stable and resistant to disturbances, indicating that reclamation and ploughing decrease the stability and resilience of microbial communities. This may be linked to the greater biodiversity in pristine grasslands, as biodiversity often enhances ecosystem stability. Furthermore, Li et al.’s (2024) experimental results suggest that long-term fertilizer application and herbicide spraying may also contribute to weakening bacterial interactions.

In-depth analyses of the top 8 bacterial phyla in terms of abundance revealed that Firmicutes exhibited the most significant difference in abundance between treatments. They accounted for 7.03% in the cropland soils (F treatment) and 13.46% in the pristine grassland soils (CK treatment), which coincides with the results of our analysis of differences between groups. Firmicutes are key players in soil systems (Li X. et al., 2020). Bacteria in this phylum decompose organic matter to gain energy, enriching soil nutrients for other bacteria. This process generally enhances positive bacterial correlations and complementary effects, aligning with our findings. Soil nutrient measurements showed higher levels in the F treatment than in CK, possibly explaining the reduced positive bacterial correlations in F. Previous work by Li et al. (2023) suggests that richer soil nutrients could reduce the need for bacterial interactions during nutrient cycling. By analyzing the changes in soil bacterial symbiotic networks under different treatments, we can gain a deeper understanding of bacterial interrelationships and the dynamics within soil ecosystems under different land use patterns. These findings improve our understanding of soil bacterial communities’ structure, providing scientific guidance for managing and protecting soil ecosystems.

### 4.3 Assembly of bacterial community under changing land use patterns

The composition and diversity of soil bacterial communities are central drivers of ecosystem functioning, and their formation is regulated by a combination of deterministic and stochastic processes (Chang et al., 2025). Deterministic processes include environmental conditions, species competition, and different survival needs (Feng et al., 2025). Stochastic processes involve limitations in dispersal, ecological drift, and random variations in birth and death rates (Burns et al., 2016). To analyze these community assembly mechanisms, researchers use models such as the Neutral Community Model (NCM) and null models. In this study, we applied NCM—a framework focusing on stochastic processes like species migration—to investigate bacterial community assembly (Trinh et al., 2025). The results suggest that soil bacterial communities in the F treatment are more influenced by stochastic processes than those in the CK treatment, as shown by Figure 6. This aligns with the higher *α*-diversity in F-treated soils, likely due to enhanced bacterial dispersal capabilities. It also illustrates the limited impact of external environmental pressures on the assembly of bacterial communities. Bacterial communities that are strongly influenced by stochastic processes are usually characterized by faster bacterial renewal and higher oxygen utilization capacity. These patterns are consistent with previous findings suggesting that minor environmental changes favor stochastic assembly, while major disturbances trigger deterministic processes (Yu et al., 2022).

In this study, the soil nutrient content of the F treatment was higher than that of CK, but the difference was not significant, which led to the result that the bacterial community assembly of the F treatment was more affected by stochastic processes. Intensive and continuous ploughing and other anthropogenic practices in F introduce more disturbances than in CK, leading to a greater influence of stochastic factors in F’s community assembly. In addition to this, when analyzing the microbial network structure we found that both positive and negative cohesion were higher in the CK treatment than in the F treatment, and that high cohesion usually represents more interactions between bacteria, and that these interactions may have made the assembly of the bacterial community in the F treatment more affected by stochastic processes (Chase and Myers, 2011). This study emphasizes the dynamic balance between stochastic and deterministic processes in microbial community assemblages, which varies according to specific ecosystems and research content. Soil bacterial communities under both treatments were more affected by stochastic processes, but there was a significant difference in the ratio of stochastic to deterministic processes between the two treatments, and analyses of the reasons for this variability will help us to better understand bacterial community assemblages under different land uses.

## 5 Conclusion

Our study reveals the effects of land use change on soil bacterial communities in the black soil area of the Songnen Plain, Northeast China, and provides important insights for related ecological management. We found that reclamation significantly altered soil physicochemical properties as well as bacterial diversity patterns in terms of α-diversity, *β*-diversity, and community stability, where the relative abundance of dominant genera, such as *Sphingomonas* and *Pseudarthrobacter*, was elevated in the cropland. Random forest model showed genera such as *Rhodomicrobium* and *Amycolatopsis*, whose dynamics were correlated with changes in land use practices. Co-occurrence network analyses showed that reclamation led to a reduction in network complexity, a weakening of the ability of bacteria to interact with each other, and a reduction in positive and negative network cohesion. Community assembly analyses showed that bacterial community construction in both treatments was dominated by stochastic processes, and the croplands treatment was more affected by stochastic processes. The results revealed that the sustainable use of black soil resources needs to limit the threshold of anthropogenic disturbance to protect the stability of soil bacterial communities and their ability to interact with each other. The study has important practical value for maintaining the sustainability of black soil ecosystems.

## Data Availability

The original contributions presented in the study are publicly available. This data can be found here: https://www.ncbi.nlm.nih.gov/bioproject/PRJNA1304265/.

## Footnotes

^1^ http://gephi.github.io/

## References

[ref1] Allison S. D. Martiny J. B. H. (2008). Colloquium paper: resistance, resilience, and redundancy in microbial communities. Proc. Natl. Acad. Sci. USA 105 Suppl 1, 11512–11519. doi: 10.1073/pnas.0801925105 18695234 PMC2556421

[ref2] Atashpaz S. Khani S. Barzegari A. Barar J. Vahed S. Z. Azarbaijani R.. (2010). A robust universal method for extraction of genomic DNA from bacterial species. Mikrobiologiia 79, 562–566. doi: 10.1134/s0026261710040168 21058509

[ref3] Bao Y. Dolfing J. Chen R. Li Z. Lin X. Feng Y. (2023). Trade-off between microbial ecophysiological features regulated by soil fertility governs plant residue decomposition. Soil Tillage Res. 229:105679. doi: 10.1016/j.still.2023.105679

[ref4] Bhardwaj L. Kumar D. Singh U. P. Joshi C. G. Dubey S. K. (2024). Herbicide application impacted soil microbial community composition and biochemical properties in a flooded rice field. Sci. Total Environ. 914:169911. doi: 10.1016/j.scitotenv.2024.169911 38185156

[ref5] Bolger A. Lohse M. Usadel B. (2014). Trimmomatic: a flexible trimmer for Illumina sequence data. Bioinformatics 30, 2114–2120. doi: 10.1093/bioinformatics/btu170, PMID: 24695404 PMC4103590

[ref6] Borer E. Risch A. (2024). Planning for the future: grasslands, herbivores, and nature-based solutions. J. Ecol. 112, 2442–2450. doi: 10.1111/1365-2745.14323

[ref7] Burns A. R. Stephens W. Z. Stagaman K. Wong S. Rawls J. F. Guillemin K.. (2016). Contribution of neutral processes to the assembly of gut microbial communities in the zebrafish over host development. ISME J. 10, 655–664. doi: 10.1038/ismej.2015.142, PMID: 26296066 PMC4817674

[ref8] Chai J. Wang X. Liu X. Li C. Han J. Yao T. (2023). Inoculation of cold-adapted microbial consortium screened from alpine meadows promotes the growth of mixed grasses by changing soil properties and enzyme activity. Rhizosphere 28:100782. doi: 10.1016/j.rhisph.2023.100782

[ref10] Chang C. Hu E. Tang X. Ye S. Zhao D. Qu Z.. (2025). Assembly of soil multitrophic community regulates multifunctionality via multifaceted biotic factors in subtropical ecosystems. Environ. Int. 195:109272. doi: 10.1016/j.envint.2025.109272 39805170

[ref11] Chase J. M. Myers J. A. (2011). Disentangling the importance of ecological niches from stochastic processes across scales. Philos. Trans. R. Soc. B Biol. Sci. 366, 2351–2363. doi: 10.1098/rstb.2011.0063, PMID: 21768151 PMC3130433

[ref12] Chen W. Ren K. Isabwe A. Chen H. Liu M. Yang J. (2019). Stochastic processes shape microeukaryotic community assembly in a subtropical river across wet and dry seasons. Microbiome 7:138. doi: 10.1186/s40168-019-0749-8, PMID: 31640783 PMC6806580

[ref13] Chen Y. Shi W. Aihemaitijiang G. Zhang F. Zhang J. Zhang Y.. (2025). Hyperspectral inversion of heavy metal content in farmland soil under conservation tillage of black soils. Sci. Rep. 15:354. doi: 10.1038/s41598-024-83479-0, PMID: 39747359 PMC11696877

[ref14] Chen P. Xie Y. Ren X. Cheng C. Wei X. (2024). Spatial variation of soil organic carbon density in the black soil region of Northeast China under the influence of erosion and deposition. J. Clean. Prod. 475:143616. doi: 10.1016/j.jclepro.2024.143616

[ref16] Coller E. Cestaro A. Zanzotti R. Bertoldi D. Pindo M. Larger S.. (2019). Microbiome of vineyard soils is shaped by geography and management. Microbiome 7:140. doi: 10.1186/s40168-019-0758-7, PMID: 31699155 PMC6839268

[ref17] Conners E. Rengasamy K. Ranaivoarisoa T. Bose A. (2024). The phototrophic purple non-sulfur bacteria *Rhodomicrobium* spp. are novel chassis for bioplastic production. Microb. Biotechnol. 17:e14552. doi: 10.1111/1751-7915.14552, PMID: 39163151 PMC11334908

[ref18] D’Acunto L. Semmartin M. Ghersa C. (2016). Uncultivated margins are source of soil microbial diversity in an agricultural landscape. Agric. Ecosyst. Environ. 220, 1–7. doi: 10.1016/j.agee.2015.12.032

[ref19] de León G. Grima P. Tort-Martorell X. (2011). Comparison of normal probability plots and dot plots in judging the significance of effects in two level factorial designs. J. Appl. Stat. 38, 161–174. doi: 10.1080/02664760903301143

[ref20] Feng C. Lu J. Liu T. Shi X. Zhao S. Lv C.. (2025). Microbial community dynamics in shallow-water grass-type lakes: habitat succession of microbial ecological assembly and coexistence mechanisms. Ecotoxicol. Environ. Saf. 291:117819. doi: 10.1016/j.ecoenv.2025.117819 39908866

[ref21] Gopalakrishnan S. Srinivas V. Naresh N. Alekhya G. Sharma R. (2019). Exploiting plant growth-promoting *Amycolatopsis* sp. for bio-control of charcoal rot of sorghum (*Sorghum bicolor* L.) caused by *Macrophomina phaseolina* (Tassi) Goid. Arch. Phytopathol. Plant Protect. 52, 543–559. doi: 10.1080/03235408.2018.1553472

[ref22] Gustavsson E. Zhang D. Reynolds R. Garcia-Ruiz S. Ryten M. (2022). Ggtranscript: an R package for the visualization and interpretation of transcript isoforms using ggplot2. Bioinformatics 38, 3844–3846. doi: 10.1093/bioinformatics/btac409, PMID: 35751589 PMC9344834

[ref23] Heidrich V. Karlovsky P. Beule L. (2021). “SRS” R package and “q2-SRS” QIIME 2 plugin: normalization of microbiome data using scaling with ranked subsampling (SRS). Appl. Sci. 11:11473. doi: 10.3390/app112311473

[ref24] Heredia-Acuña C. Semchenko M. De Vries F. (2023). Root litter decomposition is suppressed in species mixtures and in the presence of living roots. J. Ecol. 111, 2519–2531. doi: 10.1111/1365-2745.14207, PMID: 38550391 PMC10976660

[ref25] Hermans S. Buckley H. Case B. Curran-Cournane F. Taylor M. Lear G. (2017). Bacteria as emerging indicators of soil condition. Appl. Environ. Microbiol. 83:e02826-16. doi: 10.1128/AEM.02826-16, PMID: 27793827 PMC5165110

[ref26] Hernandez D. J. David A. S. Menges E. S. Searcy C. A. Afkhami M. E. (2021). Environmental stress destabilizes microbial networks. ISME J. 15, 1722–1734. doi: 10.1038/s41396-020-00882-x, PMID: 33452480 PMC8163744

[ref27] Hoffman A. Holzer M. (2019). Invasion fronts on graphs: the fisher-KPP equation on homogeneous trees and Erdos-Reyni graphs. arXiv 24, 671–694. doi: 10.3934/dcdsb.2018202

[ref28] Jiang M. Song Y. Kanwar M. Ahammed G. Shao S. Zhou J. (2021). Phytonanotechnology applications in modern agriculture. J. Nanobiotechnol. 19:430. doi: 10.1186/s12951-021-01176-w, PMID: 34930275 PMC8686395

[ref29] Jiang Y. Zhang Y. Zhong X. Jiang J. Zhu F. Huang S.. (2025). Metabolism of *Penicillium oxalicum*-mediated microbial community reconstructed by nitrogen improves stable aggregates formation in bauxite residue: a field-scale demonstration. J. Clean. Prod. 493:144963. doi: 10.1016/j.jclepro.2025.144963

[ref30] Ju W. Li J. Yu W. Zhang R. (2016). iGraph: an incremental data processing system for dynamic graph. Front. Comp. Sci. 10, 462–476. doi: 10.1007/s11704-016-5485-7

[ref31] Kafi S. Karimi E. Motlagh M. Amini Z. Mohammadi A. Sadeghi A. (2021). Isolation and identification of *Amycolatopsis* sp. strain 1119 with potential to improve cucumber fruit yield and induce plant defense responses in commercial greenhouse. Plant Soil 468, 125–145. doi: 10.1007/s11104-021-05097-3

[ref32] Lal R. (2004). Soil carbon sequestration to mitigate climate change. Geoderma 123, 1–22. doi: 10.1016/j.geoderma.2004.01.032

[ref33] Li Z. Deng X. Jin G. Mohmmed A. Arowolo A. (2020). Tradeoffs between agricultural production and ecosystem services: a case study in Zhangye, Northwest China. Sci. Total Environ. 707:136032. doi: 10.1016/j.scitotenv.2019.136032, PMID: 31972910

[ref34] Li X. Ding L. Yuan H. Li X. Zhu Y. (2020). Identification of potential electrotrophic microbial community in paddy soils by enrichment of microbial electrolysis cell biocathodes. J. Environ. Sci. 87, 411–420. doi: 10.1016/j.jes.2019.07.016, PMID: 31791514

[ref35] Li R. Dörfler U. Munch J. C. Schroll R. (2017). Enhanced degradation of isoproturon in an agricultural soil by a *Sphingomonas* sp. strain and a microbial consortium. Chemosphere 168, 1169–1176. doi: 10.1016/j.chemosphere.2016.10.084, PMID: 27817898

[ref36] Li Z.-X. Li J.-H. Zhang Q. Lu J.-J. Gao C.-H. Jin D.-S.. (2024). Response and assembly process of soil microbial communities under different reclamation measures. Environ. Sci. 45, 7326–7336. doi: 10.13227/j.hjkx.202312247 39628196

[ref37] Li S. Wang C. Yang S. Chen W. Li G. Luo W.. (2023). Determining the contribution of microbiome complexity to the soil nutrient heterogeneity of fertile islands in a desert ecosystem. Sci. Total Environ. 857:159355. doi: 10.1016/j.scitotenv.2022.159355, PMID: 36240927

[ref38] Liu X. Burras C. Kravchenko Y. Duran A. Huffman T. Morras H.. (2012). Overview of mollisols in the world: distribution, land use and management. Can. J. Soil Sci. 92, 383–402. doi: 10.4141/CJSS2010-058

[ref39] Liu Z. Cao S. Sun Z. Wang H. Qu S. Lei N.. (2021). Tillage effects on soil properties and crop yield after land reclamation. Sci. Rep. 11:4611. doi: 10.1038/s41598-021-84191-z, PMID: 33633306 PMC7907092

[ref40] Liu Y. Yang Z. Zhang L. Wan H. Deng F. Zhao Z.. (2024). Characteristics of bacterial community structure and function in artificial soil prepared using red mud and Phosphogypsum. Microorganisms 12:1886. doi: 10.3390/microorganisms12091886, PMID: 39338562 PMC11434353

[ref41] Liu Y. Zhai Q. Lv J. Wu Y. Liu X. Zhang H.. (2025). Construction of a fusant bacterial strain simultaneously degrading atrazine and acetochlor and its application in soil bioremediation. Sci. Total Environ. 962:178478. doi: 10.1016/j.scitotenv.2025.178478, PMID: 39818196

[ref42] Liu H. Zhang W. Wang K. (2009). Effect of reclamation on soil properties of zonal and intrazonal grasslands in agro-pastoral ecotone. Trans. Chin. Soc. Agric. Eng. 25, 272–277. doi: 10.3969/j.issn.1002-6819.2009.10.049

[ref43] Meng X. Liang X. Wang P. Ren L. (2024). Effect of thermophilic bacterial complex agents on synergistic humification of carbon and nitrogen during lignocellulose-rich kitchen waste composting. J. Environ. Manag. 370:122799. doi: 10.1016/j.jenvman.2024.122799, PMID: 39393336

[ref44] Mizukami-Murata S. Sakakibara F. Fujita K. Fukuda M. Kuramata M. Takagi K. (2016). Detoxification of hydroxylated polychlorobiphenyls by *Sphingomonas* sp. strain N-9 isolated from forest soil. Chemosphere 165, 173–182. doi: 10.1016/j.chemosphere.2016.08.127, PMID: 27649311

[ref45] Mutlu H. Gökpinar F. Gökpinar E. Gül H. Güven G. (2017). A new computational approach test for one-way ANOVA under heteroscedasticity. Commun. Stat. Theory Methods 46, 8236–8256. doi: 10.1080/03610926.2016.1177082

[ref46] Netsianda A. Mhangara P. (2025). Aboveground biomass estimation in a grassland ecosystem using Sentinel-2 satellite imagery and machine learning algorithms. Environ. Monit. Assess. 197:138. doi: 10.1007/s10661-024-13610-1, PMID: 39762565 PMC11703881

[ref47] Niu B. Paulson J. N. Zheng X. Kolter R. (2017). Simplified and representative bacterial community of maize roots Microbiology. Proc. Natl. Acad. Sci. USA 114, E2450–E2459. doi: 10.1073/pnas.1616148114, PMID: 28275097 PMC5373366

[ref48] Nuruzzaman M. Bahar M. M. Naidu R. (2025). Diffuse soil pollution from agriculture: impacts and remediation. Sci. Total Environ. 962:178398. doi: 10.1016/j.scitotenv.2025.178398 39808904

[ref49] Qiu J. Bai J. Wang Y. Zhai Y. Zhang X. Xu Y.. (2024). Cadmium contamination decreased bacterial network complexity and stability in coastal reclamation areas. J. Hazard. Mater. 476:134896. doi: 10.1016/j.jhazmat.2024.134896, PMID: 38909464

[ref50] Sagar S. Singh A. Bala J. Chauhan R. Kumar R. Bhatia R.. (2024). Insights into cow dung-based bioformulations for sustainable plant health and disease management in organic and natural farming system: a review. J. Soil Sci. Plant Nutr. 24, 30–53. doi: 10.1007/s42729-023-01558-z

[ref51] Sahu P. K. Singh D. P. Prabha R. Meena K. K. Abhilash P. C. (2019). Connecting microbial capabilities with the soil and plant health: options for agricultural sustainability. Ecol. Indic. 105, 601–612. doi: 10.1016/j.ecolind.2018.05.084

[ref52] Santana-Martinez J. C. Aguirre-Monroy A. M. MacKenzie M. D. Lanoil B. D. (2024). The impact of reclamation and vegetation removal on compositional and functional attributes of soil microbial communities in the Athabasca Oil Sands region. Appl. Soil Ecol. 198:105368. doi: 10.1016/j.apsoil.2024.105368

[ref53] Schloss P. Gevers D. Westcott S. (2011). Reducing the effects of PCR amplification and sequencing artifacts on 16S rRNA-based studies. PLoS One 6:e27310. doi: 10.1371/journal.pone.0027310, PMID: 22194782 PMC3237409

[ref54] Schreckinger J. Mutz M. Gessner M. Gerull L. Frossard A. (2025). Fundamental shifts in soil and sediment microbial communities and functions during 10 year of early catchment succession. Soil Biol. Biochem. 203. doi: 10.1016/j.soilbio.2025.109713

[ref55] Schut A. Reymann W. (2023). Towards a better understanding of soil nutrient dynamics and P and K uptake. Plant Soil 492, 687–707. doi: 10.1007/s11104-023-06209-x

[ref56] Sentis A. Haegeman B. Montoya J. M. (2021). Stoichiometric constraints modulate temperature and nutrient effects on biomass distribution and community stability. Oikos 2022:oik.08601. doi: 10.1111/oik.08601, PMID: 36644620 PMC7614052

[ref57] Taguchi Y.-H. Oono Y. (2005). Relational patterns of gene expression via non-metric multidimensional scaling analysis. Bioinf (Oxf) 21, 730–740. doi: 10.1093/bioinformatics/bti067, PMID: 15509613

[ref58] Tang J. Xie Y. Cheng H. Liu G. (2025). Impact of farmland landscape characteristics on gully erosion in the black soil region of Northeast China. Catena 249:108623. doi: 10.1016/j.catena.2024.108623

[ref59] Tong Y. Liu J. Li X. Sun J. Herzberger A. Wei D.. (2017). Cropping system conversion led to organic carbon change in China’s Mollisols regions. Sci. Rep. 7:18064. doi: 10.1038/s41598-017-18270-5, PMID: 29273775 PMC5741738

[ref60] Trinh H. P. Lee S.-H. Nguyen T. V. Park H.-D. (2025). Contribution of the microbial community to operational stability in an anammox reactor: neutral theory and functional redundancy perspectives. Bioresour. Technol. 419:132029. doi: 10.1016/j.biortech.2024.132029, PMID: 39740752

[ref61] Van Midden C. Harris J. Shaw L. Sizmur T. Pawlett M. (2023). The impact of anaerobic digestate on soil life: a review. Appl. Soil Ecol. 191:105066. doi: 10.1016/j.apsoil.2023.105066, PMID: 40769517

[ref62] Visioli G. Sanangelantoni A. Vamerali T. Dal Cortivo C. Blandino M. (2018). 16S rDNA profiling to reveal the influence of seed-applied biostimulants on the rhizosphere of young maize plants. Molecules 23:1461. doi: 10.3390/molecules23061461 29914131 PMC6100521

[ref63] Wang C. Kuzyakov Y. (2024). Mechanisms and implications of bacterial-fungal competition for soil resources. ISME J. 18:wrae073. doi: 10.1093/ismejo/wrae073 38691428 PMC11104273

[ref9001] Wang M. Frey B. Li D. Liu X. Chen C. Liu Y.. (2024). Effects of organic nitrogen addition on soil microbial community assembly patterns in the Sanjiang Plain wetlands, northeastern China. Appl. Soil Ecol. 204:14. doi: 10.1016/j.apsoil.2024.105685

[ref64] Wang J. Mi W. Song P. Xie H. Zhu L. Wang J. H. (2018). Cultivation ages effect on soil physicochemical properties and heavy metal accumulation in greenhouse soils. Chin. Geogr. Sci. 28, 717–726. doi: 10.1007/s11769-018-0980-4

[ref65] Wang Y. Zou Q. (2024). Deciphering microbial adaptation in the rhizosphere: insights into niche preference, functional profiles, and cross-kingdom co-occurrences. Microb. Ecol. 87:74. doi: 10.1007/s00248-024-02390-3, PMID: 38771320 PMC11108897

[ref66] Xiao E. Wang Y. Xiao T. Sun W. Deng J. Jiang S.. (2021). Microbial community responses to land-use types and its ecological roles in mining area. Sci. Total Environ. 775:145753. doi: 10.1016/j.scitotenv.2021.145753

[ref67] Xing K. Lu W. Huang Q. Wu J. Shang H. Wang Q.. (2024). Soil eDNA biomonitoring reveals changes in multitrophic biodiversity and ecological health of agroecosystems. Environ. Res. 262:119931. doi: 10.1016/j.envres.2024.119931, PMID: 39260717

[ref68] Xun W. Liu Y. Li W. Ren Y. Xiong W. Xu Z.. (2021). Specialized metabolic functions of keystone taxa sustain soil microbiome stability. Microbiome 9:35. doi: 10.1186/s40168-020-00985-9, PMID: 33517892 PMC7849160

[ref69] Yu Y. Shi Y. Li M. Wang C. Zhang L. Sun Z.. (2022). Land-use type strongly affects soil microbial community assembly process and inter-kingdom co-occurrence pattern in a floodplain ecosystem. Appl. Soil Ecol. 179:104574. doi: 10.1016/j.apsoil.2022.104574

[ref70] Zhang C. Lei S. Wu H. Liao L. Wang X. Zhang L.. (2024). Simplified microbial network reduced microbial structure stability and soil functionality in alpine grassland along a natural aridity gradient. Soil Biol. Biochem. 191:109366. doi: 10.1016/j.soilbio.2024.109366

[ref71] Zhang M. W. Liu H. Zhang M. A. Yang H. Jin Y. Han Y.. (2021). Mapping soil organic matter and analyzing the prediction accuracy of typical cropland soil types on the northern Songnen plain. Remote Sens 13:5162. doi: 10.3390/rs13245162

[ref72] Zhang H. Meltzer P. Davis S. (2013). RCircos: an R package for Circos 2D track plots. BMC Bioinformatics 14:244. doi: 10.1186/1471-2105-14-244 23937229 PMC3765848

[ref73] Zhang D. Wang C. Li X. Yang X. Zhao L. Liu L.. (2018). Linking plant ecological stoichiometry with soil nutrient and bacterial communities in apple orchards. Appl. Soil Ecol. 126, 1–10. doi: 10.1016/j.apsoil.2017.12.017

[ref74] Zhao Y. Wang M. Hu S. Zhang X. Ouyang Z. Zhang G.. (2018). Economics- and policy-driven organic carbon input enhancement dominates soil organic carbon accumulation in Chinese croplands. Proc. Natl. Acad. Sci. USA 115, 4045–4050. doi: 10.1073/pnas.1700292114, PMID: 29666318 PMC5910801

[ref75] Zhao R. Zhang X. Jin D. Zhu X. Ji L. Gao B.. (2025). Anammox sludge granulation and synergy with comammox: a critical review. J. Environ. Chem. Eng. 13:115300. doi: 10.1016/j.jece.2024.115300, PMID: 40769517

[ref76] Zheng F. Gao J. Tang M. Zhou T. Zhu D. Yang X.. (2024). Urbanization reduces the stability of soil microbial community by reshaping the diversity and network complexity. Chemosphere 364:143177. doi: 10.1016/j.chemosphere.2024.143177, PMID: 39182733

[ref77] Zheng B. Su L. Hui N. Jumpponen A. Kotze D. J. Lu C.. (2024). Urbanisation shapes microbial community composition and functional attributes more so than vegetation type in urban greenspaces across climatic zones. Soil Biol. Biochem. 191:109352. doi: 10.1016/j.soilbio.2024.109352

[ref78] Zhou Y. Liu Y. Li X. (2024). USEARCH 12: open-source software for sequencing analysis in bioinformatics and microbiome. iMeta 3:e236. doi: 10.1002/imt2.236, PMID: 39429875 PMC11487603

[ref79] Zhou Y. Selvam A. Wong J. (2014). Evaluation of humic substances during co-composting of food waste, sawdust and Chinese medicinal herbal residues. Bioresour. Technol. 168, 229–234. doi: 10.1016/j.biortech.2014.05.070, PMID: 24951275

