# Supplementary material for: Land use change has profoundly altered the process of bacterial community assembly in the northeastern black soil zone
Source: Front Microbiol. 2025 Aug 26;16:1640134. doi: 10.3389/fmicb.2025.1640134 (PMC12417421; doi:10.3389/fmicb.2025.1640134)
Supplement: Supplementary file 1 [file Data_Sheet_1.pdf]

Table S1. Soil physical and chemical properties of farmland (F) and pristine grassland (CK).

Treatments	pH	EC (μS/cm)	NO_3^--N (mg/kg)	NH_4^+-N (mg/kg)	TP (g/kg)	AP (g/kg)
F	5.7800±0.5392a	159.9511±14.1178a	3572.3097±314.7089a	257.3030±23.6082a	913.8692±83.8658a	25742.7941±2362.4194a
CK	5.4556±0.4283b	159.6889±41.7537a	3556.4643±930.7555a	243.0979±18.7553b	863.4070±66.6264b	24321.3248±1876.8020b

Table S2. Alpha diversity of soil bacterial communities in farmland (F) and pristine grassland (CK).

Treatments	Observed_species	Chao1	ACE	Shannon	Simpson	Pielou	goods_coverage
F	629.3333±86.63429a	741.4910±95.8510a	724.2276±101.2272a	5.0367±0.1902a	0.9855±0.0036a	0.7825±0.0185a	0.9943±0.0009a
CK	520.7778±36.29317b	625.9045±44.54146b	620.9766±44.3297b	4.7648±0.1480b	0.9823±0.0032a	0.7618±0.1606a	0.9947±0.0004a

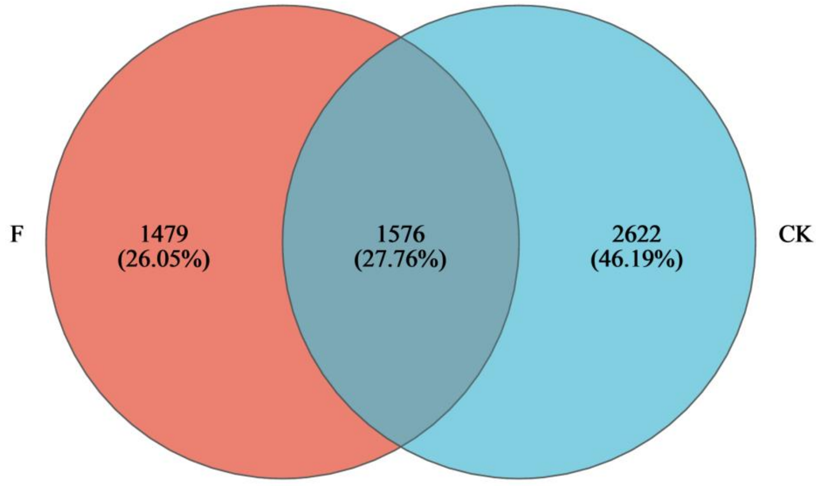

Fig. S1. Variance distribution of soil microbial communities in farmland (F) and pristine grassland (CK). Significance levels according to Monte Carlo permutation test (1000 permutations). Different colors represent different treatment conditions. Overlapping areas represent bacterial genera common to the different treatment conditions. Non-overlapping areas represent bacterial genera specific to that treatment condition.

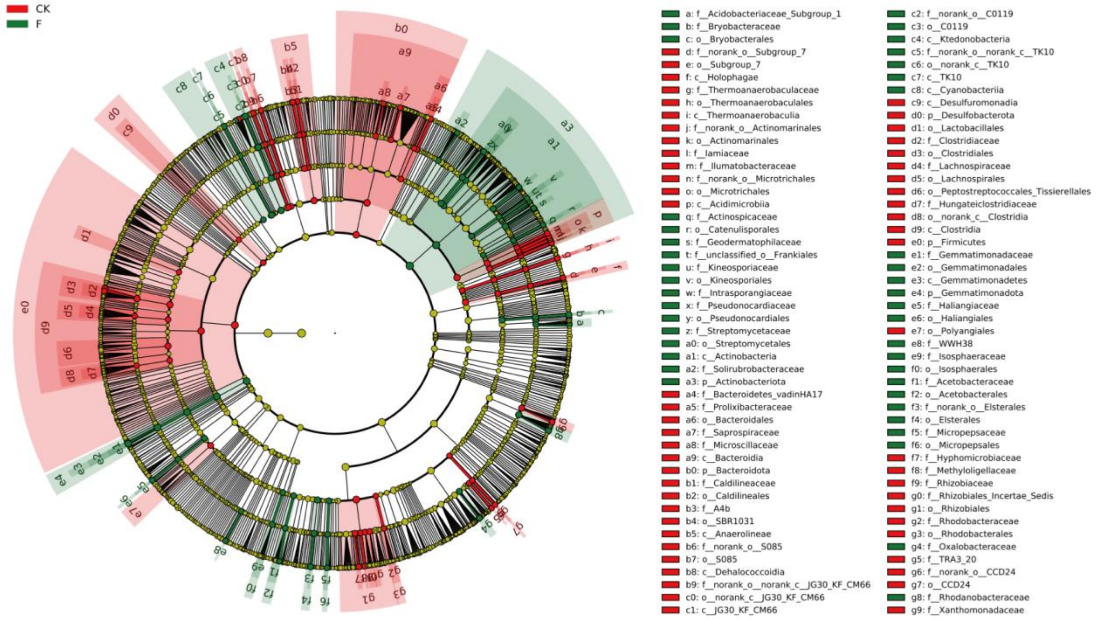

Fig. S2. LEfse analysis based on OTUs in farmland (F) and pristine grassland (CK). The circles radiating from inside to outside represent taxonomic levels from phylum to genus (or species). Each small circle at a different taxonomic level represents a classification under that level, and the size of the circle diameter is proportional to the relative abundance size. The legend shows the names of the species indicated by the letters in the figure. Hollow nodes: categorical units with no significant differences between groups. Colored nodes: taxonomic units with significant differences between different groups. The letters in the figure correspond to the legend on the right and indicate the name of the indicated species classification.
